# Feeding artery remodeling and long-term regression of flow related aneurysms following obliteration of brain arteriovenous malformations

**DOI:** 10.1016/j.bas.2026.106274

**Published:** 2026-08-07

**Authors:** Giannis Sokratous, Tamara Tajsic, Yuhan Guo, Daniel Brown, Mathew R. Guilfoyle, Adel Helmy, Rikin A. Trivedi

**Affiliations:** a Department of Clinical Neurosciences, University of Cambridge, Cambridge, UK; b Department of Neurosurgery, Addenbrooke's Hospital, Cambridge, UK; c School of Clinical Medicine, University of Cambridge, Cambridge, UK

**Keywords:** Arteriovenous malformation, Distal feeding artery, Flow aneurysms, Proximal feeding artery

## Abstract

**Introduction:**

Flow aneurysms associated with arteriovenous malformations may increase risk of hemorrhagic presentation and unfavorable outcome but their natural history and management, following treatment of the associated malformation remain uncertain.

**Research question:**

To evaluate the relationship between changes in feeding vessel caliber and flow related aneurysm size, following complete surgical resection or stereotactic radiosurgery obliteration of the arteriovenous malformation.

**Material and methods:**

A retrospective observational study was performed (2008–2017), including all patients with symptomatic arteriovenous malformation, at least one flow related, conservatively managed aneurysm and angiographically complete resection or obliteration of the malformation following treatment. Aneurysm, proximal and distal feeding artery dimensions were obtained before and after treatment. Spearman rank coefficients and linear regression analysis were used to describe any correlation, its strength and direction.

**Results:**

Eleven patients with seventeen aneurysms were eligible for inclusion, of which fourteen conservatively managed flow aneurysms were analyzed. Eight patients harboring eleven flow aneurysms underwent surgical resection, with the remaining three patients receiving radiosurgery. After excluding completely regressed aneurysms, proximal feeding artery remodeling showed moderate-to-strong correlation to aneurysm regression (ρ = 0.68, P = .021), while distal feeding artery remodeling was unrelated (ρ = 0.00, P = 1.0). Distal type IIb flow related aneurysms were more likely to regress.

**Discussion and conclusions:**

Changes in proximal feeding artery diameter may help predict fate of flow aneurysms. Our findings support incorporating proximal feeding artery remodeling into post-treatment radiological assessment. With further validation, it may represent a biomarker for treatment efficacy in patients with flow aneurysms, and help inform personalized treatment strategies.

## Highlights

• Proximal feeding artery remodeling correlates with aneurysm regression
• Distal feeding artery remodeling shows no link to aneurysm regression
• Distal aneurysms more likely to regress after arteriovenous malformation obliteration
• Proximal artery change may guide personalized imaging surveillance

## 1 Introduction

Flow aneurysms (FAs) associated with arteriovenous malformations (AVMs) are thought to increase the risk of hemorrhagic presentation and unfavorable outcome (Stapf, 2002; Westphal and Grzyska, 2000). The natural history and management of these following treatment of the associated AVM remain uncertain. The incidence varies between studies, with most reporting this to be between 10 and 20%, and this has increased in recent years due to improved diagnostic techniques such as 3D and super selective angiography (Almefty and Spetzler, 2011; da Costa et al., 2009; Flores et al., 2014; Rammos et al., 2016). Flow aneurysms are thought to form as a result of complex interactions between arterial blood flow, patient specific characteristics and genetic predisposition factors (Almefty and Spetzler, 2011; da Costa et al., 2009; Flores et al., 2014; Rammos et al., 2016).

A significant factor appears to be the disrupted hemodynamics secondary to the increased blood flow through the AVM, a process thought to result in hypertrophy of the feeding arteries, and the formation of flow related aneurysms (Alaraj et al., 2015). Wall shear stress (WSS) is a term used to describe the force applied by the flowing blood on the vessel wall, mainly the endothelium, and is a force that has been identified as a critical factor determining vessel wall and vascular remodeling (Epstein et al., 1994; Papaioannou and Stefanadis, 2005).

This complex process additionally involves disrupted endothelial signaling, abnormal cell behavior and inflammation. Histological and molecular studies demonstrate endothelial cells with increased density and impaired rearrangement, thought to arise, at least in part, from disrupted signaling pathways such as the Rbpj/Notch and Hippo/YAP. These intracellular cascade changes affect cell polarity, migration and adhesion resulting in poor vascular remodeling and the development of vascular shunts (Adhicary et al., 2023; Mahmoud et al., 2010; Neyazi et al., 2025).

Cerebral AVMs have long been described as dynamic lesions, with their feeders known to enlarge with time and regress following resection of the malformation (Alaraj et al., 2015; Hamby, 1958; Oppenheim et al., 1999). In agreement with previous reports, our recent study showed that flow related aneurysms tend to regress following treatment of the associated AVM, a finding that is significant for those flow aneurysms that are close to the nidus or distal on the feeding artery (Budohoski et al., 2021; Elhammady et al., 2013; Flores et al., 2014; Redekop et al., 1998).

In this current study, we evaluate the relationship between the changes observed in feeding vessel caliber and flow related aneurysm size, following complete surgical resection or stereotactic radiosurgery (SRS) obliteration of the AVM.

## 2 Methods

A retrospective data collection from electronic patient records and angiography imaging between 2008 and 2017 was performed at a single tertiary neurosciences center. This study was approved by the local audit committee. Due to anonymized data, no ethics approval was required. We included all patients that presented with a symptomatic arteriovenous malformation, were found to have at least one flow related aneurysm on pre-operative angiography and had post-treatment angiographic confirmation that the arteriovenous malformation had been completely resected by microsurgery or obliterated by radiosurgery but retained at least one flow related aneurysm managed conservatively with surveillance.

Intracranial aneurysms associated with brain AVMs were classified according to the Redekop classification (Redekop et al., 1998). Aneurysms located on an artery not feeding the AVM nidus were classified as unrelated (type I). Aneurysms located on the feeding artery were classified as flow related and further divided into: (type IIa) located on the main trunk of the intracranial vessel up to its primary bifurcation; (type IIb) located past the primary bifurcation of the main vessel; and (type III) intranidal.

We collected quantitative data of the flow aneurysm dimensions (width, height), and of the proximal feeding artery (PFA) and distal feeding arteries (DFA). Values were recorded in millimeters before and after treatment of the AVM both as absolute values and as the measured change (Δ) following treatment.

Measurements were taken using our dedicated diagnostic software measuring tool (McKesson Radiology Solutions, Murfreesboro, Tennessee, USA), by two independent researchers and average values were documented.

Clinical characterization of the cohort was performed to contextualise the radiological findings. Extracted outcomes included peri-procedural complications, focal sensory and motor neurological deficits, modified Rankin Scale (mRS) at discharge and mRS at last available follow-up following AVM treatment. Analysis was performed at a patient level, and is summarized using descriptive statistics. The institution's electronic medical record was the sole data source.

Following our previous findings, we hypothesized that there would be a relative lesser degree of feeding vessel remodeling in those patients in whom, post-AVM obliteration, the flow aneurysm failed to regress. Spearman rank correlation coefficients were determined for all available imaging variables. Simple linear regression analysis was used to estimate the strength and direction of association between changes observed on the FAs and each arterial remodeling variable, for both proximal and distal feeding arteries. Statistical significance was defined as P < .05. Analyses were conducted in Python (version 3.14, Python Software Foundation) with figures produced using the matplotlib library.

We analyzed the data using two complementary approaches. In the primary analysis, aneurysms that completely regressed following treatment were assigned a post-treatment area of zero, thereby preserving all eligible observations and reflecting complete anatomical resolution. Because complete regression precludes assessment of the magnitude of further size reduction, we additionally performed a secondary exploratory analysis restricted to aneurysms that remained measurable after treatment. This complementary analysis was intended to evaluate the relationship between feeding artery remodeling and the degree of residual aneurysm regression and was interpreted as hypothesis-generating.

## 3 Results

Data from a total of eleven patients with a mean age of 43 years and harboring 17 aneurysms, were eligible for inclusion. The baseline characteristics are summarized in Table 1. One of these aneurysms was contralateral to the AVM and unrelated, another was intranidal and treated as part of the AVM and a third one was treated on presentation as it had ruptured. The remaining fourteen aneurysms were flow related (IIa and IIb) and included in this study (Table 1).

**Table 1:** Patient specific demographics and characteristics.

Patient	Gender	Age	Presentation	Spetzler-Ponce (SM)	GCS	Treatment
*Patient 1*	F	57	Hemorrhage	A(I)	15	Surgery
*Patient 2*	M	29	Hemorrhage	B(III)	15	Surgery
*Patient 3*	F	63	Hemorrhage	A(I)	15	Surgery
*Patient 4*	M	52	Hemorrhage	A(I)	15	Surgery
*Patient 5*	F	40	Hemorrhage	A(I)	15	Surgery
*Patient 6*	F	25	Hemorrhage	A(I)	14	Surgery
*Patient 7*	F	51	Hemorrhage	B(III)	15	Surgery
*Patient 8*	F	63	Hemorrhage	A (II)	11	Surgery
*Patient 9*	F	26	Seizures	B(III)	14	Radiosurgery
*Patient 10*	F	43	Seizures	B(III)	15	Radiosurgery
*Patient 11*	F	26	Seizures	A(I)	15	Radiosurgery

Eight patients presented acutely with rupture and three presented with seizures. Glasgow Coma Scale (GCS) at presentation varied from 11 to 15.

All patients presenting with hemorrhage underwent computed tomography (CT) angiogram on admission followed by digital subtraction angiography (DSA). Patients presenting with seizures were initially investigated with magnetic resonance imaging (MRI) followed by DSA.

All patients found to have a symptomatic AVM in our department are discussed in a dedicated, regional multidisciplinary meeting (MDT) and a treatment plan is proposed. Typically, Spetzler-Martin (SM) I and II – Spetzler-Ponce (SP) A are treated surgically, SM IV and V - SP C are treated with SRS or active surveillance and those with SM III - SP B are treated with either option or combination, based on individual characteristics, age, and patient wishes (Spetzler and Martin, 1986; Spetzler and Ponce, 2011).

Patients were divided into surgical and radiosurgical groups with the former consisting of eight patients harboring eleven and the latter consisting of three patients harboring three flow related, conservatively managed aneurysms.

Six patients in the surgical group had one; one patient had two, and one had three FAs. Six patients had SP class A and two had SP class B AVMs. One patient with SP class B AVM underwent partial neo-adjuvant endovascular embolization prior to microsurgical resection. All three patients in the radiosurgery group had one FA each, two had SP class B and one had SP class A AVMs.

No intra-operative complications occurred in either the surgical or radiosurgery group. Post-operatively, one patient experienced cerebrospinal fluid leak, one developed a resection site haematoma which required evacuation, and two experienced transient neurological deficits attributed to intervention. All events occurred in the surgery group. At discharge, six patients had focal motor deficits, and two had sensory deficits. Median mRS at discharge was 2 (IQR 1.0 – 2.5).

Follow-up was available for all patients, with a median duration of 33 months (IQR 11.5–84.0). Motor deficits had improved partially or completely in five patients. Of the two patients with sensory deficits, one showed partial improvement, while the other had completely resolved. Overall functional outcome improved in the majority of patients. Median mRS at last follow-up was 1 (IQR 0–1.5), with improvement in seven patients. Only one patient had higher mRS at follow-up, though this was due to frailty related changes. Of note, the patient who developed a haematoma remained significantly neurologically impaired, with residual motor deficits and mRS 5 at last follow-up.

Timing of follow-up DSA differed between treatment modalities. Patients in the surgical group underwent post-operative DSA at a mean of 3.8 days. The FA disappeared in one and had a reduced size in the remaining cases in this group. Patients in the radiosurgery group underwent DSA at a mean 3.6 years following treatment. In two of the radiosurgery group patients, the FAs disappeared but remained unchanged in the third patient.

Five patients in the surgical group were found to have had delayed cranial imaging for unrelated reasons without any gross change in the size of the FAs when compared to the post-operative DSA. One patient had one of their aneurysms treated electively 3 years after treatment of the AVM.

**Fig. 1:**
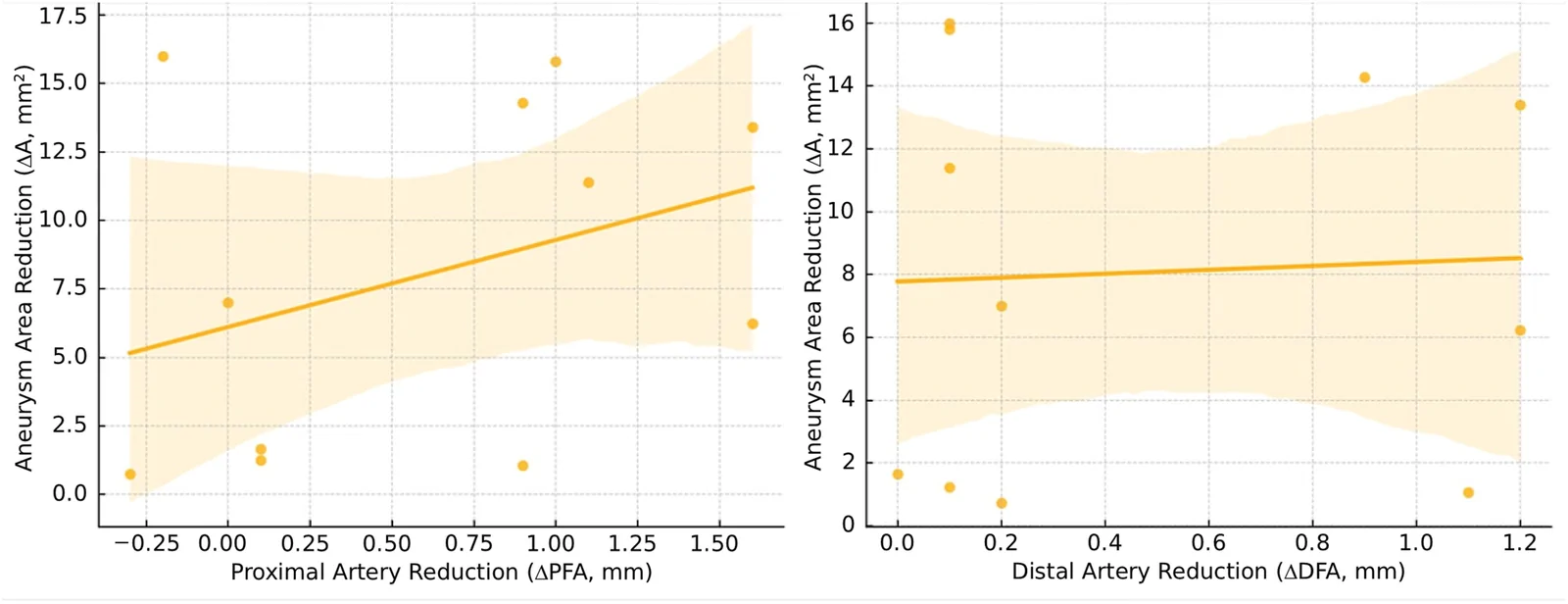
Weak correlation between ΔA and ΔPFA with slightly negative, weak correlation between ΔDFA and ΔA when aneurysms that had fully regressed were assigned the numerical value zero (0). A, aneurysm area; DFA, distal feeding artery; PFA, proximal feeding artery; Δ, change.

Analysis of the original dataset, where aneurysms that had fully regressed, were assigned the numerical value zero, showed weak correlation between ΔPFA and ΔA (ρ = 0.28, P = .332) and slightly negative, weak correlation between ΔDFA and ΔA (ρ = −0.15, P = .609) (Fig. 1). Moderate correlation was seen between ΔPFA and ΔDFA (ρ = 0.55, P = .042), with DFA remodeling being greater than that observed in the PFA.

**Fig. 2:**
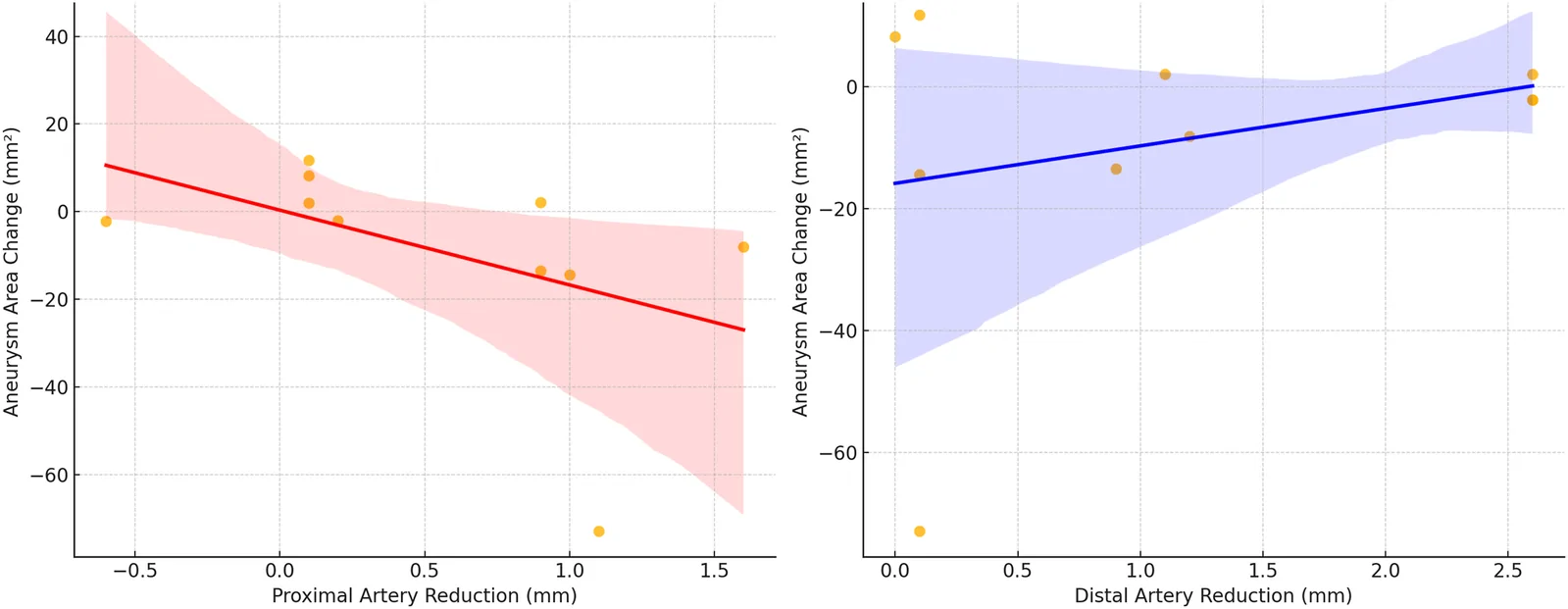
Moderate to strong correlation between ΔPFA and ΔA with weak between ΔDFA and ΔA when aneurysms that had fully regressed were not included. A, aneurysm area; DFA, distal feeding artery; PFA, proximal feeding artery; Δ, change.

When fully regressed aneurysms were excluded, the correlation between ΔPFA and ΔA was moderate to strong (ρ = 0.68, P = .021) but remained weak between ΔDFA and ΔA (ρ = 0.00, P = 1.0) (Fig. 2).

**Fig. 3:**
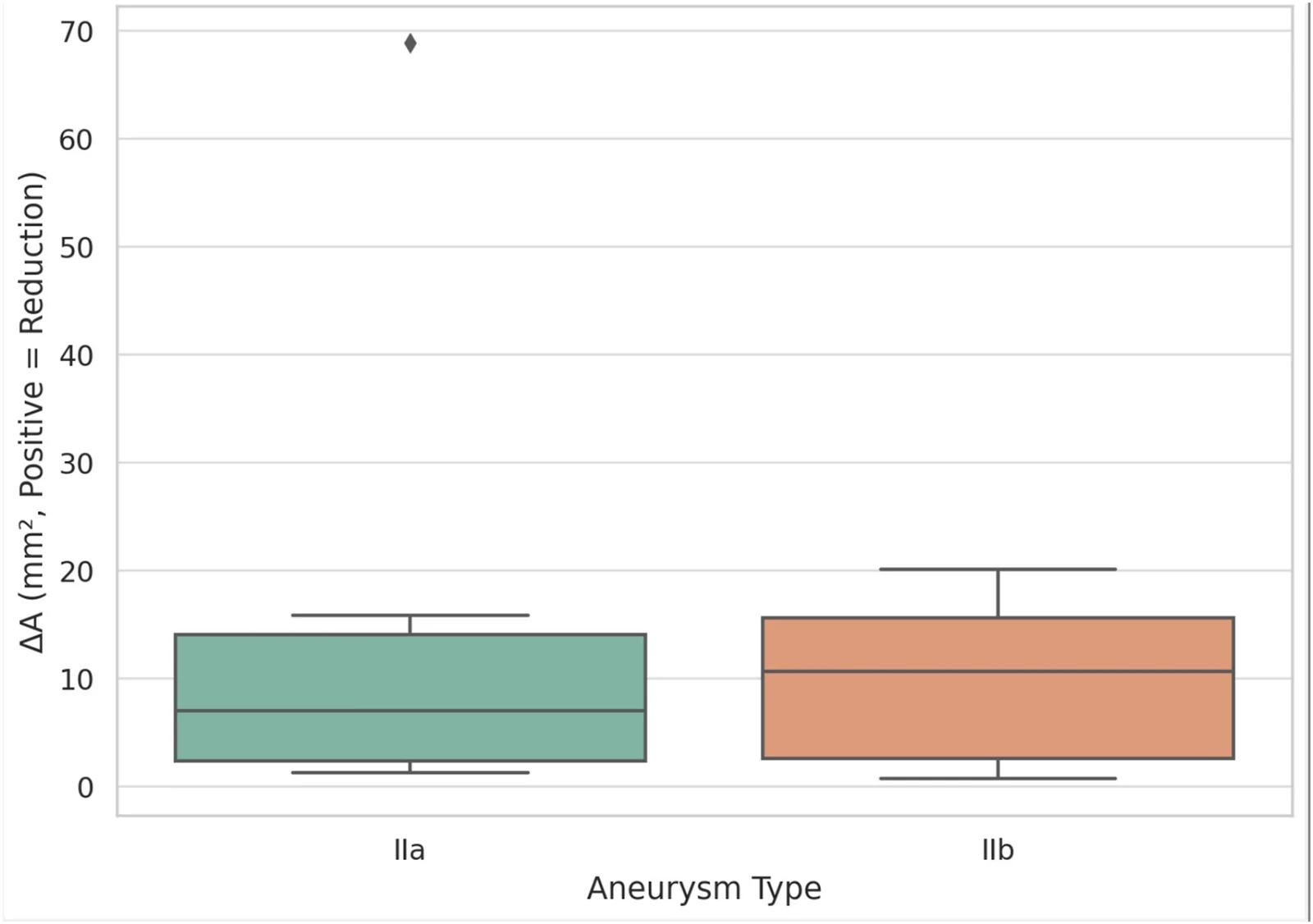
Box plot indicating that IIb aneurysms were more likely to reduce in size or resolve with large median and upper quartile ranges as several cases reached the maximum ΔA. Greater variability and lesser degree of regression was seen with IIa aneurysms. ΔA, aneurysm area reduction.

Analysis based on aneurysm type showed that type IIb aneurysms were more likely to reduce in size or resolve with large median and upper quartile ranges as several cases reached the maximum ΔA. Greater variability and lesser degree of regression was seen with IIa aneurysms (Fig. 3).

## 4 Discussion

Our findings demonstrate that changes observed in PFA diameter following obliteration of the AVM were correlated with reduction in aneurysm size. In contrast, DFA remodeling, while greater, appears to show no significant relationship with flow aneurysm regression.

Additionally, type IIb aneurysms seem more likely to regress or disappear following obliteration, compared to type IIa FAs. This finding is consistent with previous reports indicating a greater likelihood of aneurysms closer to the nidus disappearing or reducing in size, following AVM obliteration (Budohoski et al., 2021; D'Aliberti et al., 2015). This anatomical gradient aligns with the expected proximal-to-distal dissipation of wall shear stress (WSS) and offers insight into the mechanistic locus of reverse remodeling in flow related aneurysms.

The relationship shown between proximal remodeling and aneurysm size is physiologically intuitive. Proximal vessels are more directly exposed to the elevated flow associated with AVM shunting, making them more susceptible to shear stress and wall tension. Successful AVM obliteration normalizes these parameters, leading to structural regression—a phenomenon previously described but now supported with these findings, which infer a reduction in flow and pressures more distally and hence regression of the downstream FA (Akeret et al., 2019; Shah et al., 2017).

Of note, the observed lack of relationship between DFA caliber change and aneurysm size suggests that distal vessel caliber does not necessarily influence aneurysm dynamics, as the majority of this flow dynamic change has occurred more proximally. This underscores the importance of contextual hemodynamics: DFA changes may reflect downstream autoregulation or secondary adaptations rather than primary flow redistribution from the AVM.

These findings form a coherent and physiological model whereby proximal flow unloading**,** when significant enough, induces aneurysm involution/regression—especially in anatomically susceptible aneurysm subtypes.

Interestingly, two out of the three aneurysms that completely regressed, were treated with SRS. The small number of cases precludes meaningful statistical analysis, but does raise the question of possible additional albeit unintended endoproliferation within the SRS field of the FA itself. The length of time between treatment and first post-treatment angiogram was longer in the SRS group, but this alone cannot explain this phenomenon, as a number of patients in the surgical group underwent brain imaging in the subsequent years to brain AVM obliteration, though no dedicated vascular imaging was performed on those occasions.

We now know that multiple factors other than blood flow, play a significant role in vascular remodeling. Inflammation, abnormal endothelial cell proliferation and migration and dysfunctional cell-cell junctions have now been described as at least contributing factors in AVM formation, with a number of molecular pathways, such as the bone morphogenetic protein (BMP) signaling thought to be responsible (Arthur and Roman, 2022; Banerjee et al., 2023; Edgar et al., 2022). Whether radiotherapy has a role to play in reversing or improving some of these factors and in extend increasing the likelihood of size reduction in parent vessel and flow aneurysm remains to be described. It is however likely that slower and stepwise regression of the AVM following treatment with SRS could allow for more controlled, natural reversal of such factors allowing for higher chances of aneurysm regression.

The inclusion of clinical outcome data provides characterization of the cohort relative to the radiological findings. Despite the occurrence of deficits, clinical outcomes were largely stable or improved. Most patients demonstrated partial or complete improvements in motor and sensory deficits, with stable or improved mRS at last available follow-up. Given the small sample size, meaningful conclusions between treatment modalities cannot be drawn. Nevertheless, the results provide insight into the practical relevance of the radiological findings.

Unlike previous studies where strictly descriptive methods were employed, we have shown a correlation between PFA remodeling and the fate of flow related aneurysms. It may thus be reasonable to include measurements of ΔPFA diameter during post-treatment surveillance imaging, particularly for type IIb aneurysms, which often present clinicians with anxiety about possible future expansion or rupture and need for longer term surveillance. However, given the limitations of the present study, ΔPFA should not yet be used in isolation. If further validated in larger cohorts, ΔPFA may help inform personalized follow-up intervals, surveillance protocols and early intervention if necessary.

### 4.1 Limitations

Our study is not without limitations. The small sample size, while typical for AVM-FA series, restricts statistical power and limits generalizability. For said reason we employed Spearman's rank correlation coefficient (ρ) as our primary correlation measure. This decision is methodologically justified as it does not assume normality, making it suitable for skewed and non-linear data (both likely in this study given the heterogeneous aneurysm profiles), it allows detection of relationships even in the absence of linearity, providing superior sensitivity in small sample populations while being less sensitive to outliers.

Additionally, assigning disappearing aneurysms the numerical value zero, had the expected result of reducing the correlation between ΔPFA and ΔA as the relationships between values were more diluted, the rank continuity needed for Spearman ρ was broken, increasing heterogeneity and reducing statistical power. The exploratory analysis excluding completely regressed aneurysms should be interpreted cautiously, as restricting the analysis according to post-treatment aneurysm status may introduce selection bias. Accordingly, the complete-cohort analysis remains the primary analysis of the study. Furthermore, imaging-derived measurements were mostly based on 2D DSA and assumed elliptical aneurysm morphology; 3D volumetrics or computational flow modeling would yield more granular insights.

Lastly, the retrospective design precludes causal inference and cannot account for confounding factors such as systemic hemodynamic variability, AVM location, or staged interventions. Importantly, the cohort lacked standardized timing for follow-up imaging, introducing possible bias from differential remodeling kinetics.

## 5 Conclusion

Within the limitations of this study, we have shown that the fate of flow related aneurysms may be associated with changes in diameter of the proximal feeding arteries. Our findings support incorporating proximal feeding artery remodeling into post-treatment radiological assessment. If further validated in larger cohorts, ΔPFA could serve as a biomarker for treatment efficacy in FA-bearing AVM patients, with the potential to guide personalized follow-up intervals, surveillance protocols and early intervention if necessary.

## Author contribution

GS, DB, MRG, AH and RAT conceived and designed the study. GS, YG and TT prepared and collected the data. GS analyzed the data and drafted the initial manuscript. GS and YG prepared the figures. All authors reviewed and revised the manuscript. All authors approved the final version of the manuscript.

## Ethics approval

The project was registered and approved by the hospital audit committee (18/03/2025). Formal research ethics not required under local policy.

## Funding

No funding was received for conducting this study.

## Declaration of competing interest

The authors declare that they have no known competing financial interests or personal relationships that could have appeared to influence the work reported in this paper.

## Data Availability

Data available on request from the corresponding author subject to institutional approvals.
